# Specific Susceptibility to COVID-19 in Adults with Down Syndrome

**DOI:** 10.1007/s12017-021-08651-5

**Published:** 2021-03-04

**Authors:** Tomer Illouz, Arya Biragyn, Milana Frenkel-Morgenstern, Orly Weissberg, Alessandro Gorohovski, Eugene Merzon, Ilan Green, Florencia Iulita, Lisi Flores-Aguilar, Mara Dierssen, Ilario De Toma, Hefziba Lifshitz, Stylianos E. Antonarakis, Eugene Yu, Yann Herault, Marie-Claude Potier, Alexandra Botté, Randall Roper, Benjamin Sredni, Ronit Sarid, Jacqueline London, William Mobley, Andre Strydom, Eitan Okun

**Affiliations:** 1 The Leslie and Susan Gonda Multidisciplinary Brain Research Center, Bar-Ilan University, 5290002 Ramat-Gan, Israel; 2 The Paul Feder Laboratory On Alzheimer’s Disease Research, Bar-Ilan University, 5290002 Ramat-Gan, Israel; 3 Laboratory of Molecular Biology and Immunology, NIA, Baltimore, MD 21224 USA; 4 Cancer Genomics and BioComputing of Complex Diseases Lab, Azrieli Faculty of Medicine, Bar-Ilan University, Safed, Israel; 5 Leumit Health Services, Department of Family Medicine, Sackler School of Medicine, Tel Aviv University, Tel Aviv-Yafo, Israel; 6 Sant Pau Memory Unit, Department of Neurology, Hospital de La Santa Creu I Sant Pau, Barcelona, Spain; 7 Biomedical Research Institute Sant Pau, Universitat Autònoma de Barcelona, Barcelona, Spain; 8 Center of Biomedical Investigation Network for Neurodegenerative Diseases (CIBERNED), Madrid, Spain; 9 Alzheimer-Down Unit, Fundación Catalana Síndrome de Down, Barcelona, Spain; 10 Department of Anatomy and Cell Biology, McGill University, Montreal, Canada; 11 Center for Genomic Regulation, The Barcelona Institute for Science and Technology, Barcelona, Spain; 12 University Pompeu Fabra, Barcelona, Spain; 13 Biomedical Research Networking Center for Rare Diseases (CIBERER), Barcelona, Spain; 14 Cellular & Systems Neurobiology, Systems Biology Program, The Barcelona Institute of Science and Technology, Centre for Genomic Regulation (CRG), Dr. Aiguader 88, 08003 Barcelona, Spain; 15 Universitat Pompeu Fabra (UPF), Barcelona, Spain; 16 Centro de Investigación Biomédica en Red de Enfermedades Raras (CIBERER), Madrid, Spain; 17 School of Education, Bar-Ilan University, 5290002 Ramat-Gan, Israel; 18 Department of Genetic Medicine and Development, University of Geneva, 1211 Geneva, Switzerland; 19 Medigenome, Swiss Institute of Genomic Medicine, 1207 Geneva, Switzerland; 20 iGE3 Institute of Genetics and Genomics of Geneva, 1211 Geneva, Switzerland; 21 The Children’s Guild Foundation Down Syndrome Research Program, Genetics and Genomics Program and Department of Cancer Genetics and Genomics, Roswell Park Comprehensive Cancer Center, Buffalo, NY USA; 22 Genetics, Genomics and Bioinformatics Program, State University of New York At Buffalo, Buffalo, NY USA; 23 Université de Strasbourg, CNRS, INSERM, Institut de Génétique Biologie Moléculaire Et Cellulaire, IGBMC–UMR, 7104 - Inserm U1258, 1 rue Laurent Fries, ILLKIRCH, 67404 Cedex, France; 24 Paris Brain Institute (ICM), CNRS UMR7225, INSERM U1127, Sorbonne Université, Hôpital de La Pitié-Salpêtrière, Paris, France; 25 Department of Biology, Indiana University-Purdue University Indianapolis, Indianapolis, USA; 26 The Mina and Everard Goodman Faculty of Life Sciences, Bar-Ilan University, 5290002 Ramat-Gan, Israel; 27 Université de Paris, BFA, UMR 8251, CNRS, 7501 Paris, France; 28 Department of Neurosciences, University of California, San Diego, USA; 29 Department of Forensic and Neurodevelopmental Sciences, Institute of Psychiatry Psychology and Neuroscience, King’s College London, 16 De Crespigny Park, London, SE5 8AF UK; 30 South London and Maudsley NHS Foundation Trust, London, UK

**Keywords:** COVID-19, Down syndrome, Immune dysregulation, SARS-CoV-2, Vaccine

## Abstract

The current SARS-CoV-2 outbreak, which causes COVID-19, is particularly devastating for individuals with chronic medical conditions, in particular those with Down Syndrome (DS) who often exhibit a higher prevalence of respiratory tract infections, immune dysregulation and potential complications. The incidence of Alzheimer’s disease (AD) is much higher in DS than in the general population, possibly increasing further the risk of COVID-19 infection and its complications. Here we provide a biological overview with regard to specific susceptibility of individuals with DS to SARS-CoV-2 infection as well as data from a recent survey on the prevalence of COVID-19 among them. We see an urgent need to protect people with DS, especially those with AD, from COVID-19 and future pandemics and focus on developing protective measures, which also include interventions by health systems worldwide for reducing the negative social effects of long-term isolation and increased periods of hospitalization.

**Supplementary Information:**

The online version contains supplementary material available at 10.1007/s12017-021-08651-5.

## Overview

**Fig. 1:**
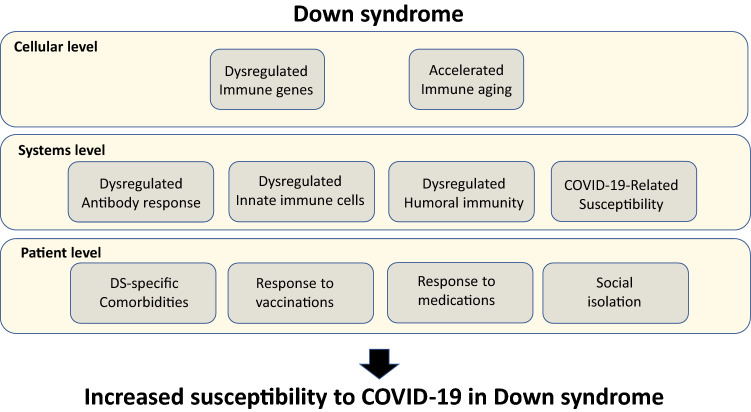
Susceptibility factors to COVID-19 in Down’s syndrome. Top bar; Cellular-level susceptibility factors. Middle bar; System-level susceptibility factors. Lower bar; patient-level susceptibility factors. All these factors culminate in increased susceptibility of individuals with DS to SARS-Cov-2

The current COVID-19 pandemic, caused by the SARS-CoV-2 virus, is of widespread concern for the elderly people and populations at risk due to co-existing medical conditions, including cardiovascular diseases, obesity, and diabetes. Individuals with Down syndrome (DS) appear to have a significantly higher risk of developing severe symptoms of infectious diseases, including those related to COVID-19, because of their complex trisomy and consequent numerous immune impairments, which render them susceptible to infections. Chromosome 21 (Chr21), triplicated in individuals with DS, includes genes directly involved in SARS-CoV-2 entry into cells, potentially augmenting COVID-19-specific susceptibility (Fig. 1). Clinically, these immune dysregulations often result in ineffective vaccination against infectious diseases. COVID-19-related risks, such as anatomical airway features, which enhance airway-related infections, congenital heart disease, hypertension, obesity, and diabetes, are prevalent in individuals with DS. The immune anomalies of individuals with DS suggest that any COVID-19-directed therapy developed for the general population requires careful testing prior to implementation in the clinic. Not least important is the additional aspect of social isolation experienced by numerous at-risk individuals in general and individuals with DS in particular (Fig. 1). Individuals with DS are often dependent on regular schedules, which, upon interruption, can impair their mental health. Measures to avert the implications of social isolation in this vulnerable population, therefore, need to be taken into consideration. This complex picture calls for an immediate reorganization towards better preparedness for future pandemics in vulnerable populations, including DS.

## Down Syndrome–Prevalence and Lifespan

**Fig. 2:**
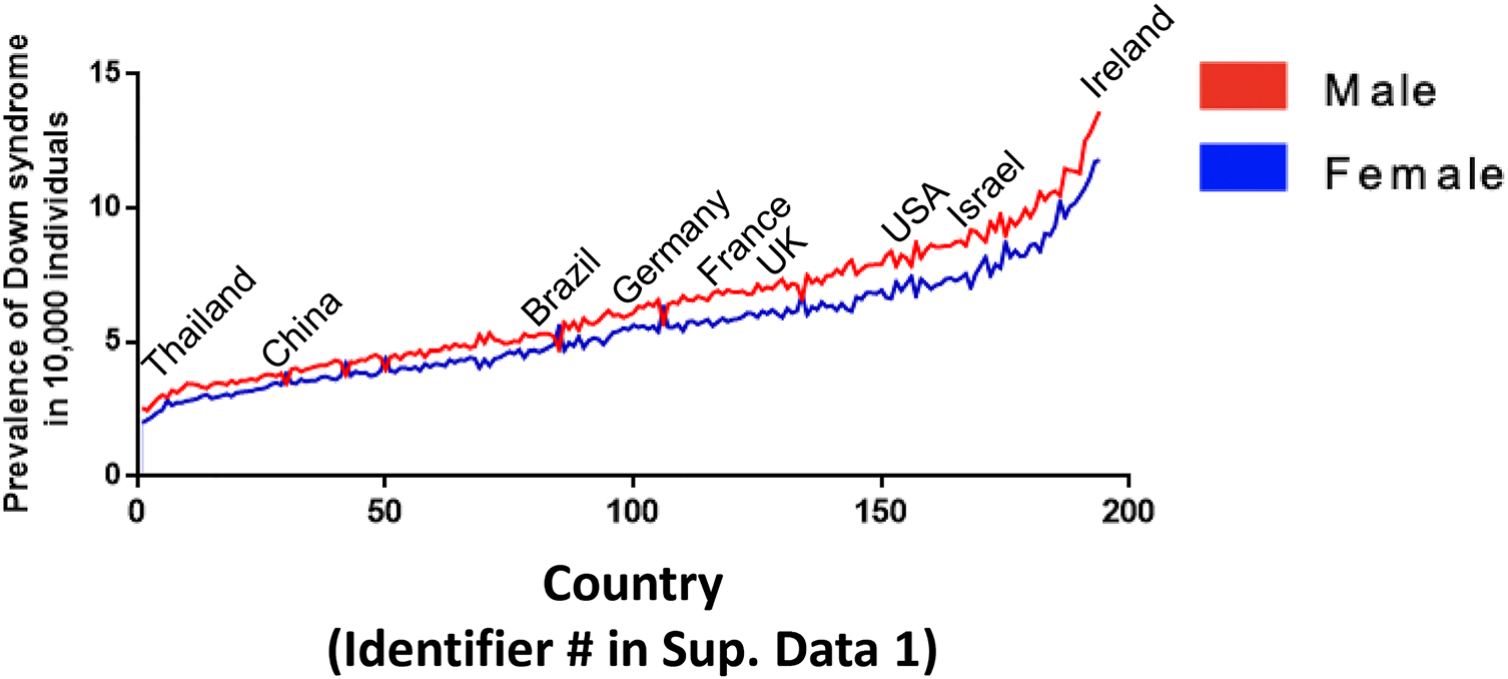
Worldwide prevalence of DS. Data collected from the Global Health Data Exchange (GHDx; http://ghdx.healthdata.org/) were used to calculate the total worldwide prevalence of DS according to sex in 194 countries

DS is the most common genetic disorder of cognition; essentially, all those with DS demonstrate intellectual disability (ID), though of variable degree. Chr21 contains approximately 233 protein-coding genes, some of which, like amyloid precursor protein (*APP)* and dual-specificity tyrosine phosphorylation-regulated kinase 1A (*DYRK1A)*, have been linked to an Alzheimer’s disease (AD)-like phenotype. DS occurs in all populations with an average worldwide prevalence of 3.3–6.7 per 10,000 individuals that varies between countries (Antonarakis et al. 2020) (Fig. 2, Supplementary Data 1).

In the 1940s, the average life expectancy for people with DS was 12 years (Penrose, 1949). With advances and increased access to medical care and early surgical repair of congenital heart malformations, individuals with DS currently have an average life expectancy of 60 years (Bittles & Glasson, 2004). The risk for mortality in younger people with DS is increased mainly because of leukemia and respiratory infections. As a result of increased life expectancy, a greater number of individuals with DS now exhibit age-related diseases, such as AD—the principal cause of death in the elderly with DS, which may increase their risk for emerging pathogens such as SARS-CoV-2. A significant vulnerability to infections impacts individuals with DS after the age of 50 years, especially those with certain comorbidities, like seizures and age-related onset of dementia (Guffroy et al. 2019).

## Comorbidities in DS as Risk Factors for Emerging Infectious Diseases

Obesity, increasing age, male sex, hypertension, and diabetes are risk factors associated with increased mortality from COVID-19 in the general population (Brenner et al. 2020; George et al. 2020; Palaiodimos et al. 2020) and are also present in DS, combining to increase susceptibility to infectious diseases including SARS-CoV-2. Certain anatomical features contribute a short stature, a flat and wide face with a flat nasal ridge, slanted eyes, a short neck, and a protruding tongue due to a small orofacial cavity and a relatively large tongue (Kanamori et al. 2000). These features impact the airway tract and may contribute to the increase in obstructive sleep apnea and upper airway obstruction found in 50% of those with DS (Horne et al. 2019; Marcus et al. 1991). Intervention with respiratory support may therefore prevent COVID-19-related consequences of sleep-disordered breathing (Trucco et al. 2018).

Pulmonary hypertension is diagnosed in 13% of those with DS (Berger et al. 2012). This condition and developmental lung anomalies (Cooney & Thurlbeck, 1982) may combine to increase the risk of COVID-19 in people with DS (Bush et al. 2020), due to reduced oxygen capacity and supply.

Obesity, due to lower thyroid and a lower metabolic rate, and ID co-occur significantly more often than in the general population. Obesity is associated with decreased expiratory reserve volume and functional capacity. Abdominal obesity compromises pulmonary function in immobilized patients by decreased diaphragmatic excursion and reducing ventilation efficacy. Obesity is also associated with higher levels of inflammatory cytokines, contributing to increased morbidity associated with obesity in COVID-19 infections (Dietz & Santos-Burgoa, 2020). Obesity is also associated with obstructive sleep apnea, another very common condition in individuals with DS, which, if left untreated, can lead to hypertension, heart failure and sudden death.

Additionally, the frequency of congenital heart disease varies from 40 to 54% in DS (Freeman et al. 1998). The cardiac defects are usually treated by pediatric cardiac surgery at an early age (Jensen et al. 2014). As for those with congenital heart diseases, individuals with DS are known to have a higher risk for complications with viral illnesses, including respiratory syncytial virus and influenza (Alsaied et al. 2020; Weijerman et al. 2010).

## Prevalence of Infectious Diseases and Associated Hospitalizations in Down Syndrome

Bacteria such as *Streptococcus pneumonia* are the major cause of pneumonia in individuals with DS (Verstegen et al. 2014), while viruses such as *influenza virus, respiratory syncytial virus* (*RSV*), and *parainfluenza virus* account for most respiratory infections in DS (Chan et al. 2017). RSV, a double-stranded RNA (dsRNA) virus, is the primary cause of severe lower respiratory tract infections (LRI) in young children (< 5 years) (Beckhaus & Castro-Rodriguez, 2018; Perez-Padilla et al. 2010). Children with DS are at risk for RSV infections (Beckhaus & Castro-Rodriguez, 2018; Mitra et al. 2018), especially those who have chronic lung disease or congenital heart disease.

Following the outbreak of the H1NI 2009 pandemic in Mexico, a study found a 16-fold increase in hospitalization, an eightfold increase in endotracheal intubation, and a more than 300-fold increase in death in individuals with DS in Mexico (Perez-Padilla et al. 2010). Moreover, DS patients were significantly younger as compared with affected individuals of the general population. Thus, compared with the general population, DS is associated with increased susceptibility to bacterial and viral infections at a younger age, resulting in worse outcomes, higher hospitalization rates, longer hospital stays, and higher mortality rates.

These findings point to respiratory infections as a significant source of increased morbidity and mortality in children with DS and suggest that this will apply to infection with COVID-19 in both children and adults with DS.

A recent study, reported an increased severity of COVID-19 in hospitalized patients with DS (Malle et al. 2020). This dual-center study of 7246 patients hospitalized with COVID-19 in the city of New York, assessed the hospitalization rates, clinical characteristics, and outcomes. The researchers identified 12 patients with DS, and reported that their average age was ten years younger than patients without DS. The incident of severe disease was higher in those patients, specifically an increased incident of sepsis and mechanical ventilation. Moreover, a description of the clinical course in four COVID-19 patients with DS in Belgium, revealed severe illness in three of the four cases with fatal outcome in one patient (De Cauwer & Spaepen, 2020).

## Immune Dysregulation and Aging in Down Syndrome vis-a-vis Vaccines

Individuals with DS have reduced lymphocyte numbers (Huggard et al. 2018; MacLean et al. 2018), thought to be a result of increased thymic dysfunction and apoptosis of immune cells (Schoch et al. 2017; Zampieri et al. 2014). The thymus is smaller in DS infants than in non-DS infants and exhibits structural abnormalities (Carsetti et al. 2015; Eijsvoogel et al. 2017), arguing that immune dysregulation and immunodeficiency are integral to the syndrome (Carsetti et al. 2015; Huggard et al. 2018). The selective cell-mediated immunodeficiency and impaired antibody response to pathogens in DS can be associated with the mortality associated with pneumonia and other respiratory diseases in children with DS (Chaushu et al. 2002; Costa-Carvalho et al. 2006).

Type I interferons (IFNs) are a group of proteins that help regulate the activity of the immune system. DS is characterized by constitutive IFN-I signaling; this may be a major cause of suboptimal vaccine responses in people with DS. This raises the question as to whether SARS-CoV-2 vaccines shown effective in otherwise healthy young hosts will prove effective in people with DS. It is premature to conclude that the DS population will fail to respond to SARS-CoV-2 vaccines that are effective in the general population. However, this should be proactively explored through studies in which strategies that block high levels of IFN-I signaling or upregulation of IFN receptors are tested. Alternatively, vaccine responses can be improved using immune modulators, such as interleukin (IL)-21, or activating downstream signaling effectors of IL21, such as the STAT3 pathway, known to contribute to long-lived antibody-secreting cells in humans (Avery et al. 2010).

Initial response to vaccines is generally adequate in individuals with DS but with lower mean titers and a need for more frequent booster immunizations compared to individuals without DS (Eijsvoogel et al. 2017). Many of these immunological alterations are age related and can be envisioned as within the spectrum of early senescence of the immune system, which is typically seen in the elderly (da Rosa Utiyama et al. 2008; Trotta et al. 2011). Indeed, premature aging in multiple body tissues is evident in DS (Zigman, 2013). Specifically, molecular aging in DS is more pronounced in individuals with DS that experience dementia compared with individuals with DS without apparent dementia. For example, telomeres are chromosome ends consisting of highly conserved TTAGGG repeats whose number progressively reduced with aging (Shay, 2018). Immune T cell lymphocyte cultures from people with DS and AD-related dementia have shorter telomeres than T-lymphocytes from age- and sex-matched people with DS with no dementia (Jenkins et al. 2008, 2010), a phenomenon that precedes the late stages of dementia in DS (Jenkins et al. 2012).

## Specific Predisposition to Severe COVID-19 in DS

Cell attachment and entry is a critical step in the process of viral infection. Of particular relevance to DS is the TMPRSS2 gene, located within Chr21 (De Cauwer, 2020; Paoloni-Giacobino et al. 1997). TMPRSS2 is a protease involved in SARS-CoV-2 entry into cells via priming of S protein; TMPRSS2 overexpression might result in a higher number of infected cells that could potentially augment COVID-19 susceptibility in DS. It was recently reported that a TMPRSS2 inhibitor, approved for clinical use, blocked entry of SARS-2-S to lung cells (Hoffmann et al. 2020), therefore might constitute a treatment option for patients with DS. Furthermore, viruses, including SARS-CoV, enter cells by hijacking mechanisms, like phagocytosis via the endocytosis pathways (Cataldo et al. 2008; Inoue et al. 2007). Dysregulation of endocytosis in DS is likely a function of an increased dose of several endosomal pathway-related genes that map to Chr21, including *APP, SYNJ1 (Synaptojanin-1), ITSN1 (Intersectin-1), and possibly RCAN1 (Regulator of calcineurin 1*) (Botte & Potier, 2020; Patel et al. 2015). Studies focused on neurons showed that APP, known to mediate dementia in DS, is involved in altered trafficking of viral material and modified pH-dependent fusion of viruses with an endosomal membrane in DS (Botte & Potier, 2020; Jiang et al. 2016; Kim et al. 2016).

Adults with DS exhibit elevated levels of the proinflammatory cytokines IL-6 and TNF-α (Altable & de la Serna, 2021), along with hyperactivity of interferon signaling, possibly due to IFNAR1, IFNAR2, IFNGR2 and IL10RB overexpression (Espinosa, 2020). Hypothetically, these dysregulations support the notion that DS individuals are at increased risk of exacerbated cytokine storm following COVID-19 infection. However, this hypothesis is not yet backed with data from humans.

Children with ID have a higher prevalence of specific comorbidities associated with poorer COVID-19 outcomes (Turk et al. 2020). No specific data on COVID-19 prevalence in pediatric patients with DS is currently available. Nevertheless, several case studies were recently described in such patients (Alsahabi et al. 2021; Kantar et al. 2020; Newman et al. 2021; Pontes et al. 2020). Out of the total of 10 cases presented in these publications, 3 (33%) were admitted to the ICU, 2 (20%) required intubation and none had died.

Given this and related factors discussed above, individuals with DS may be at higher risk of SARS-CoV-2 infection and poorer clinical outcomes.

## Vaccination Strategies

The majority of vaccination strategies in the race to control the COVID-19 aim to induce neutralizing antibodies to the SARS-CoV-2 by targeting its Spike (S) antigen and its binding to angiotensin converting enzyme 2 (ACE2), which serves as the primary mechanisms by which the virus enters cells. Elderly individuals, people with type-2-diabetes, morbidly obese individuals, and individuals with DS are in general poor responders to vaccines (Corretger, 2014). Two concerns attend considerations of vaccine development in the context of DS. COVID-19 vaccines may be challenged in their ability to elicit long-term protection in DS. Due to suboptimal primary and memory immune responses, vaccines often prove inefficient protection in DS (Costa-Carvalho et al. 2006). Moreover, even if a vaccine generates an adaptive immune response, it may induce persistent, even life-long, overactivation of innate immune cells in DS. Indeed, antibodies against SARS-CoV-2 antigens could exacerbate an already heightened immune response. Antibody-dependent enhancement of infection can also occur due to innate immune cell dysregulation, impaired destruction of phagocytosed immune complexes, or/and altered expression of Fc receptors, which recognize Fc fragment of antibodies and mediate downstream signaling. Achieving optimal responses in DS should be seen as an important research topic. One potential strategy would include the use of a conjugated pneumococcal vaccine, shown to induce antibodies responses in individuals with DS that are equally effective as in non-DS individuals (Kusters et al. 2013).

## Data on the Susceptibility of Individuals with DS to COVID-19

**Fig. 3:**
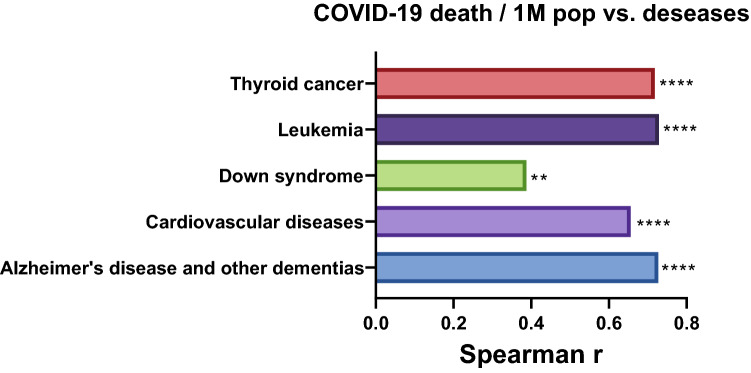
Spearman correlation between COVID-19 and different diseases. Spearman correlation between COVID-19-related deaths (normalize to 1 million) and different pathologies. A positive significant Spearman correlation was found between COVID-19 and DS, AD, cardiovascular diseases, thyroid cancer and leukemia

For an initial assessment, data on the prevalence of DS per country, as well as death rates from different diseases in 61 countries, were collected from the Global Health Data Exchange (GHDx) (http://ghdx.healthdata.org/, Sep 2020). COVID-19-related data were obtained from the WORLDOMETER database (https://www.worldometers.info/, Sep 2020). A significant correlation was observed between COVID-19-related deaths (normalized to 1 million in every country) and the prevalence of DS per country (r = 0.39, *p*-value = 0.0022, Spearman Correlation Coefficient (SCC)). By means of SCC, we observed that SARS-CoV-2 infections were positively correlated with DS-related comorbidities such as AD and dementia (SCC = 0.73, *p* < 0.0001), cardiovascular diseases (SCC = 0.66, *p* < 0.0001), leukemia (SCC = 0.72, *p* < 0.0001), and thyroid cancer (SCC = 0.71, *p* < 0.001) (Fig. 3), suggesting that DS-related comorbidities are a risk factor for COVID-19.

‘Leumit’ Health Care Services (LHS) is a health maintenance organization that covers 712,773 residents in Israel, including 570 residents with DS (0.08%). The LHS study included 115,050 subjects, (from February 1st until December 31st, 2020) aged 1 month to 106 years old, who were tested for SARS-CoV-2. Among them, 10,469 (9.1%) had at least one positive result, and 104,581 (90.9%) were negative for SARS-CoV-2 (Table 1). COVID-19 positive individuals were of lower socioeconomic status (SES) and were mostly males (Table 1). The prevalence of DS in the general population in Israel is 0.11% (GHDx, Sep 2020). The prevalence of DS individuals that were tested positive to COVID-19 in the LHS database is higher than their proportion in the general population (0.19% of all COVID-19 positive cases). We then calculated the crude odds ratio (OR) for the general population of LHS enrollees and individuals with DS that were positive for COVID-19 and we found OR = 2.33, 95% CI 1.08–3.45, (*p* = 0.023). However, we did not find significance when we compared COVID-19 positive cases in the general population with COVID-19 positive cases in individuals with DS, OR = 1.53, 95% CI 0.91–2.58, (*p* = 0.12) (Table 1). Surprisingly, chronic medical conditions such as dementia, cardiovascular disease and chronic lung disease that are considered to be risk factors for COVID-19 in previous studies, were not found as increasing the rate of infection in the LHS cohort (Table 1). It is possible that this is due to the severe social distancing imposed on all the population, and due to reduced social interaction by patients with chronic medical conditions.

**Table 1:** Demographic characteristics of subjects with DS and controls in the LHS records of SARS-CoV-2 positive with DS and SARS-CoV-2 negative (controls)

Variable		SARS-CoV-2-positive 10,469 (9.1%)	SARS-CoV-2-negative 104,581 (90.9%)	*P*-value
Mean age, (years, CI)		31.40 (30.98–31.82)	32.28 (34.12–34.43)	0.001
Social-economic status (SES) N (%)				
Low-medium		8,165 (78.0%)	66,618 (63.7%)	0.001
High-medium		1,764 (16.85%)	30,082 (28.76%)	
Missing data		539 (5.15%)	7,881 (7.54%)	
Gender N (%)				
Male		5,611 (53.6%)	50,617 (48.4%)	0.001
Female		4,858 (46.4%)	53,964 (51.6%)	
Down Syndrome		20 (0.19%)	115 (0.11%)	0.062
Obesity		1,818 (17.37%)	17,747 (16.97%)	0.028
Depression/Anxiety		342 (3.27%)	4,361 (4.17%)	0.001
Dementia		150 (1.44%)	2,802 (2.68%)	0.451
Schizophrenia		105 (1.01%)	1,307 (1.25%)	0.045
Chronic Lung Disease		768 (7.34%)	11,022 (10.54%)	0.001
Diabetes Mellitus		768 (7.34%)	9,129 (8.73%)	0.001
Hypertension		1,149 (10.98%)	16,283 (15.57%)	0.297
Ischemic Heart Disease		473 (4.52%)	7,948 (7.60%)	0.001
Heart Failure		99 (0.95%)	2,081 (1.99%)	0.124

The multiple logistic regression model for all patients tested for SARS-CoV-2 was applied to adjust for possible confounders. An association between DS and the likelihood of being tested positive for SARS-CoV-2 was significant (adjusted OR 1.64 (95% CI 0.98–2.97; *p* = 0.05) (Table 2). COVID-19 positive patients were mostly males (adjusted OR 1.20 (95% CI 1.14–1.26), with a lower-medium SES OR 2.01 (95% CI 1.88–2.15), and obese OR 1.17 (95% 1.09–1.25) (Table 2).

**Table 2:** Multivariate logistic regression for being COVID-19 positive for all patients tested for SARS-CoV-2

Variable	Adjusted Odds Ratios (95% confidence intervals)*	*P*-value
Down Syndrome	1.64 (0.98–2.97)	0.052
Male Gender	1.20 (1.14–1.26)	0.001
Low-medium- SES*	2.01 (1.88–2.15)	0.001
Obesity	1.17 (1.09–1.25)	0.001
Depression	0.98 (0.86–1.13)	0.861
Schizophrenia	0.87 (0.69–1.10)	0.268
Dementia	0.87 (0.70–1.09)	0.238
Chronic Lung Disease	0.72 (0.66–0.79)	0.001
Diabetes Mellitus	1.11 (1.01–1.24)	0.031
Hypertension	0.85 (0.77–0.94)	0.002
Ischemic Heart Disease	0.72 (0.63–0.99)	0.001
Heart Failure	0.77 (0.60–0.99)	0.044

*adjusted for sex, age, SES, and comorbidity

Individuals with DS who were positive for COVID-19 were younger compared with non-DS individuals who were positive for COVID-19 (18.48 (13.40–23.56) vs. 31.43 (31.00–31.85) years, *p*-value < 0.001). SES or gender were not a contributing factor to this difference (Table 3). Moreover, chronic lung disease was significantly more prevalent in SARS-CoV-2-positive individuals with DS (Table 3). These data suggest that individuals with DS have an increased risk for SARS-CoV-2 infection, particularly those with higher rates of chronic lung disease (*p*-value < 0.001, Table 3, and supplementary methods). Indeed, a survey that recently assessed the suceptability to COVID-19 in DS found that the mean age of COVID-19 patients with DS was lower than the general population. In addition, mortality rates were increased in individuals with DS aged 40 and above (Hüls et al. 2021).

**Table 3:** Demographic and clinical characteristics of SARS-CoV-2-positive patients with and without Down Syndrome

Variable	SARS-CoV-2-positive with DS 20 (0.19%)	SARS-CoV-2-positive without DS 10,449 (99.81%)	*P*-value
Mean age, (years, CI)	18.47 (13.39–23.55)	31.43 (31.00–31.85)	0.001
Social-economic status (SES) N (%)			
Low-medium	18 (90.0%)	8,692 (83.19%)	0.05
High-medium	2 (10.0%)	1,692 (16.19%)	
Missing data	0	54 (0.52)	
Gender N (%)			
Male	12 (60.0%)	5,601 (53.61%)	0.032
Female	8 (40.0%)	4,848 (46.39%)	
Obesity	4 (25%)	1,498 (21.81%)	0.789
Chronic Lung Disease	4 (25%)	763 (7.31%)	0.005
Heart Failure*	2 (10%)	98 (0.94%)	0.009

## Quality of Life in Individuals with DS during Social Distancing

Mention has been made about of the behavioral characteristics of people with DS that may feature in their susceptibility to infection and their physiological responses once infected. People with DS enjoy and are likely to have frequent contact with family members, caregivers, support staff, and people in the community, possibly increasing their risk of contact with infected individuals. Furthermore, they may have difficulty in understanding the importance of social distancing and wearing masks as a means of protecting against virus infection. Another important consideration is the potential impact of the pandemic on the behaviors and stress experienced by those with DS. Individuals with DS use routines and repetition to help them complete daily tasks, which are important to support their mental health. These behaviors include performing tasks at the same time and in the same way. Interrupting with these routines could, therefore, significantly affect their sense of wellbeing. Older adults with DS tend to be physically and cognitively frailer than those in the general population. As a consequence, they often live in sheltered community residences, assisted group living situations, and nursing homes. Social distancing recommendations in the context of the pandemic raise the possibility that people with DS experience increased stress, anxiety and depression and loss of social connectivity, which combine to compromise mental health concerns and induce undesirable changes in behavior. Furthermore, receiving a vaccine, as well as any other invasive medical procedure, may contribute to the stress burden experienced by individuals with DS during the pandemic. In this respect, those with DS may be representative of those in the general population who live in similar settings, especially those with dementia. As one approach to safely mitigating the deleterious effects of social distancing, the use of digital technology may provide a means by which to allow people with DS and those with memory loss and dementia to reengage with caregivers, family, and friends. Activities in a virtual space also enable availability and accessibility to services and information and improve the range of possibilities for communicating in a risk-free environment. These methods may prove useful for the elderly in general and people with DS and other sources of ID for bridging physical, geographical, and financial barriers (Caton & Chapman, 2016).

## Concluding Remarks

Here we have provided an overview of the contributing susceptibility factors to COVID-19 infection in DS, with a special focus on adults with DS. The picture that emerges from the immunological and clinical aspects of individuals with DS points to the alarming conclusion that these individuals are at increased risk for contracting specific types of infectious diseases and infection-related deaths.

There is a critical knowledge gap with respect to the underlying immune dysregulation and the specific vulnerabilities of individuals with DS to COVID-19. For example, the influence of human leukocyte antigen (HLA) gene polymorphisms on SARS susceptibility, pathogenesis, and outcome has been investigated in a number of studies. Some HLA alleles have been significantly associated with susceptibility to SARS and/or disease severity in various populations.

In addition, HLA alleles can be computationally predicted for their binding capability to SARS‐CoV‐2 peptides, predicting host susceptibility for COVID-19 disease (Ovsyannikova et al. 2020). Despite this, little is known about HLA polymorphism in the DS population. Therefore, sequencing HLA types in DS patients worldwide is needed to enable a more rapid correlation between infection rate and HLA typing in affected DS patients. This will allow mapping of protective as well as harmful HLA types in DS.

## Preparedness for Future Pandemics

Future pandemics could pose a similar or even higher risk for this susceptible population. One of the clear conclusions is that we need to increase the preparedness to future pandemics in individuals with DS in light of their increased and specific susceptibility. Better preparedness with respect to lifestyle, medical practice and basic research can be achieved mainly by forming an international network of experts in various fields, including immunology, epidemiology, neurology, and sociology. With this in mind, the Trisomy 21 Research society has established a COVID-19 task force and a stakeholders group with representatives from all major DS organizations in Europe, the U.S., and further afield.

This calls for a need to formulate steps to increase preparedness for such a pandemic by family members and clinicians. These steps should include:

1. Establishing a DS-specific worldwide database on pandemic-related symptoms and hospitalizations and response to treatment. Such a database will enable a close to a real-time understanding of the specific susceptibility of individuals with DS to pandemics and could be used or linked with existing surveillance efforts.
2. Establishing virtual social network(s) that can be activated during pandemics to support caregivers and the social welfare of individuals with DS. Such networks can partially help mitigate the adverse effects of social isolation during pandemics.
3. Promoting healthy lifestyle changes, for example lowering obesity.
4. Reducing general risk factors in individuals with DS. For example in the COVID-19 relevant medical practices include (i) improving lung health by reducing chronic/regular respiratory infections by offering influenza and pneumococcus vaccines, (ii) screening for and treating sleep apnea as well as other risk factors such as aspiration, (iii) responding early to signs of lung infection to help reduce the risk of COVID-19, and (iv) increasing surveillance and protection of high-risk older individuals, particularly those who also show signs of AD.
5. Confirming the increased risk for infection and presentation in individuals with DS when affected by COVID-19 and future pandemics.
6. Studying the clinical outcomes following COVID-19 and response to treatment in individuals with DS. The Long-term sequelae following recovery from acute COVID-19 need to be considered to understand the need for long-term management in the context of the impaired immune response in individuals with DS.
7. Establishing prior to widespread immunization whether the response to vaccines against SARS-CoV-2 in individuals with DS is similar to the response the general population.

Implementing these steps will help to support an effective response to future pandemics that is supportive not only of individuals with DS but also of family members, caregivers and clinicians, thus providing a much-needed safety net for this unique and vulnerable population.

## Supplementary Information

Below is the link to the electronic supplementary material.

Supplementary file1 (pdf 90 kb)

Supplementary file2 (xlsx 548 kb)

## Data Availability

All the data supporting the findings of this study and MATLAB codes are freely available upon request.
