# Supplementary material for: Specific Susceptibility to COVID-19 in Adults with Down Syndrome
Source: Neuromolecular Med. 2021 Mar 4;23(4):561–71. doi: 10.1007/s12017-021-08651-5 (PMC7929736; doi:10.1007/s12017-021-08651-5)
Supplement: Supplementary file 1 — Supplementary file1 (pdf 90 kb) [file 12017_2021_8651_MOESM1_ESM.pdf]

Supplementary methods

Data collection from the LHS database was performed using IBM Cognos Analytics 10.1.1 BI Report Studio software. This study was conducted using the electronic patient record (EPR) database of the LHS. All study participants have similar health insurance and similar access to health services. During each physician visit, a diagnosis is entered according to the International Classification of Diseases-9th revision. Data collected from the LHS computerized database included demographics for the entire cohort. Socioeconomic status (SES) was defined according to the subject home address, using the Israeli Central Bureau of Statistics classification, which includes 20 subgroups. Classifications one to nine were considered low-medium SES, and ten to twenty were considered medium-high SES. The data also included smoking and obesity status and other relevant medical diagnoses (including somatic and psychiatric disorders). The psychiatric diagnoses in the electronic health records, in addition to DS, included autism spectrum disorder (ASD), depressive and anxiety disorders, schizophrenia, and dementia.

The study period was from February 1st until September 22th, 2020. The study population consisted of all subjects who were LHS enrollees during the study period and underwent at least one test for SARS-CoV-2. The test used in Israel is the SARS-CoV-2 RT-PCR diagnostic panel, the AllplexTM 2019-nCoV Assay (Seegene Inc., Seoul, Republic of Korea) according to the Israeli Ministry of Health's guidelines for symptoms and close contact with SARS-CoV-2-positive subjects. The study protocol was approved by the Shamir Medical Center Review Board and the Research Committee of LHS as follows:

- The International Classification of Disease, 9th revision (ICD-9) codes were used for identification of comorbidities.
- Chronic Lung disease ICD-9 codes for asthma (493.00–493.92) and chronic bronchitis (490-496)
- Diabetes - ICD-9 codes 250.00- 250.93
- Arterial hypertension - ICD-9 codes 401- 405
- Obesity was considered as a measurement of BMI >30 m2/kg.
- Depression - ICD-9 codes: 296.20-296.25, 296.30-296.35, 300.4, 311, 309.0,309.28.
- Dementia- ICD-9 codes: 290.0 -290.4, 331.00, 331.1
- Ischemic heart disease - ICD-9 codes: 410-414.

- Congestive heart failure- ICD-9 codes: 428.0-428.9
- Socioeconomic status (SES) data was taken from the Israeli Central Bureau of Statistics classification that includes 20 sub-groups, according to the home address. Classifications one to nine were considered low-medium SES, and 10-20 high SES (17).

Statistical analysis. Data variables were analyzed using STATA 12 software (StataCorp LP, College Station, TX). Assumptions were two-sided with α of 0•05. Initial analysis compared demographic characteristics between the study groups (positive vs negative COVID-19 tests). The distributions of continuous variables and categorical variables were examined using the Kolmogorov-Smirnov non-parametric test and Fisher's exact $\chi 2$ test, respectively. Categorical data are shown in counts and percentages. Data on continuous variables with normal distribution are presented as mean and 95% confidence interval (CI). Multivariate logistic regression was used to estimate the odds ratios (OR) and 95% CI for the independent association between DS and positive PCR test for SARS-CoV-2 while controlling for potential confounders, such as age, SES, gender, and others.
